# Independent association between age- and sex-specific metabolic syndrome severity score and cardiovascular disease and mortality

**DOI:** 10.1038/s41598-023-41546-y

**Published:** 2023-09-05

**Authors:** Mohammadjavad Honarvar, Ladan Mehran, Safdar Masoumi, Sadaf Agahi, Shayesteh Khalili, Fereidoun Azizi, Atieh Amouzegar

**Affiliations:** 1 Endocrine Research Center, Research Institute for Endocrine Sciences, Shahid Beheshti University of Medical Sciences, Tehran, Islamic Republic of Iran; 2 Department of Internal Medicine, Imam Hossein Hospital, Shahid Beheshti University of Medical Sciences, Tehran, Islamic Republic of Iran

**Keywords:** Metabolic syndrome, Epidemiology

## Abstract

Traditional metabolic syndrome (MetS) criteria have several limitations, which hinder its use in clinical practice. To overcome the limitations, we investigated the association between age- and sex-specific continuous MetS severity score (cMetS-S) and cardiovascular disease (CVD) and mortality beyond MetS components in the framework of the Tehran Lipid and Glucose Study. Participants aged 20–60 years at baseline were included in the study. We excluded participants with CVD, cancer, use of corticosteroids, estimated glomerular filtration rate < 30 ml/min/1.73 m^2^, and those who were pregnant. We evaluated the association between cMetS-S with CVD and mortality over 18 years of follow-up among 8500 participants with continuous and quantile approaches using the Cox proportional hazard regression model. In addition, the model performance of cMetS-S for predicting CVD events was compared to the conventional MetS criteria. Participants with higher cMetS-S had a significantly increased risk for CVD, coronary (CHD) and non-coronary heart disease (non-CHD), and all-cause, cardiovascular, and sudden cardiac death. Independent of the confounders and MetS components, the cMetS-S had the HRs of 1.67 (95% CI 1.47–1.89), 1.60 (95% CI 1.37–1.86), and 1.88 (95% CI 1.50, 2.35) for CVD, CHD, and non-CHD events upon 1-SD increment, respectively. The risk of mortality was increased for 1-SD of cMetS-S (all-cause mortality, HR 1.24; 95% CI 1.09–1.41; CVD mortality, HR 1.72; 95% CI 1.20–2.45; sudden cardiac death, HR 1.60; 95% CI 1.03–2.49). The model fitness of cMetS-S was superior to the conventional MetS criteria in predicting CVD and mortality. The cMetS-S provided an additional risk for CVD and mortality beyond the individual MetS components. Standardized cMetS-S could be a potential universal measure to define MetS severity while considering the weighted contribution of MetS components and their variations by age, sex, and ethnicity.

## Introduction

Metabolic syndrome (MetS) is a cluster of conditions defined by five cardiometabolic risk factors: waist circumference (WC), blood pressure, fasting plasma glucose (FPG), triglyceride (TG), and high-density lipoprotein cholesterol (HDL-C). In the Middle East, nearly one in four adults are living with MetS, and more people are affected by this increasing secular trend every day^1,2^. The latest study from Iran estimated an alarming prevalence of 47.6% for MetS in the general population^3^. Atherogenic dyslipidemia and endothelial dysfunction as central features of MetS contribute to the development of atherosclerosis and cardiovascular disease (CVD)^4^. Studies have shown that MetS increases the risk of CVD incidence and all-cause mortality by 2 and 1.5 times, respectively^5^. The association of MetS with CVD makes it an effective epidemiologic tool to prevent cardiovascular-related outcomes and mortality.

The dichotomous nature of the traditional definition of MetS (presence vs. absence) has limited its use in clinical settings; the binary definition results in a loss of data regarding the degree of abnormality of the MetS components. Moreover, a minimal change in the value of the MetS components could label the individuals as having MetS or not^6^. The term pre-metabolic syndrome has been described to address such individuals with one or two MetS components that do not fit the definition of MetS^7^. A measure of MetS severity enables clinicians to monitor changes in MetS and its related adverse health events over time within populations (i.e., individuals with and without MetS).

To prevail these limitations, a few researchers have formulated a MetS severity score using confirmatory factor analysis (CFA) in different ethnicities and demonstrated its predictability for CVD^8,9^. Recently, we developed a continuous Mets severity score (cMetS-S) using CFA in the Iranian population^10^. Briefly, we defined cMetS-S by the weighted contribution of MetS components to MetS and its variation by age and sex. However, whether this score predicts incident CVD and mortality is unnoted. In this article, we intend to verify our newly developed cMetS-S by investigating its association with CVD incidence and all-cause mortality and whether the association would be independent of individual MetS components.

## Methods

### Study population

**Figure 1:**
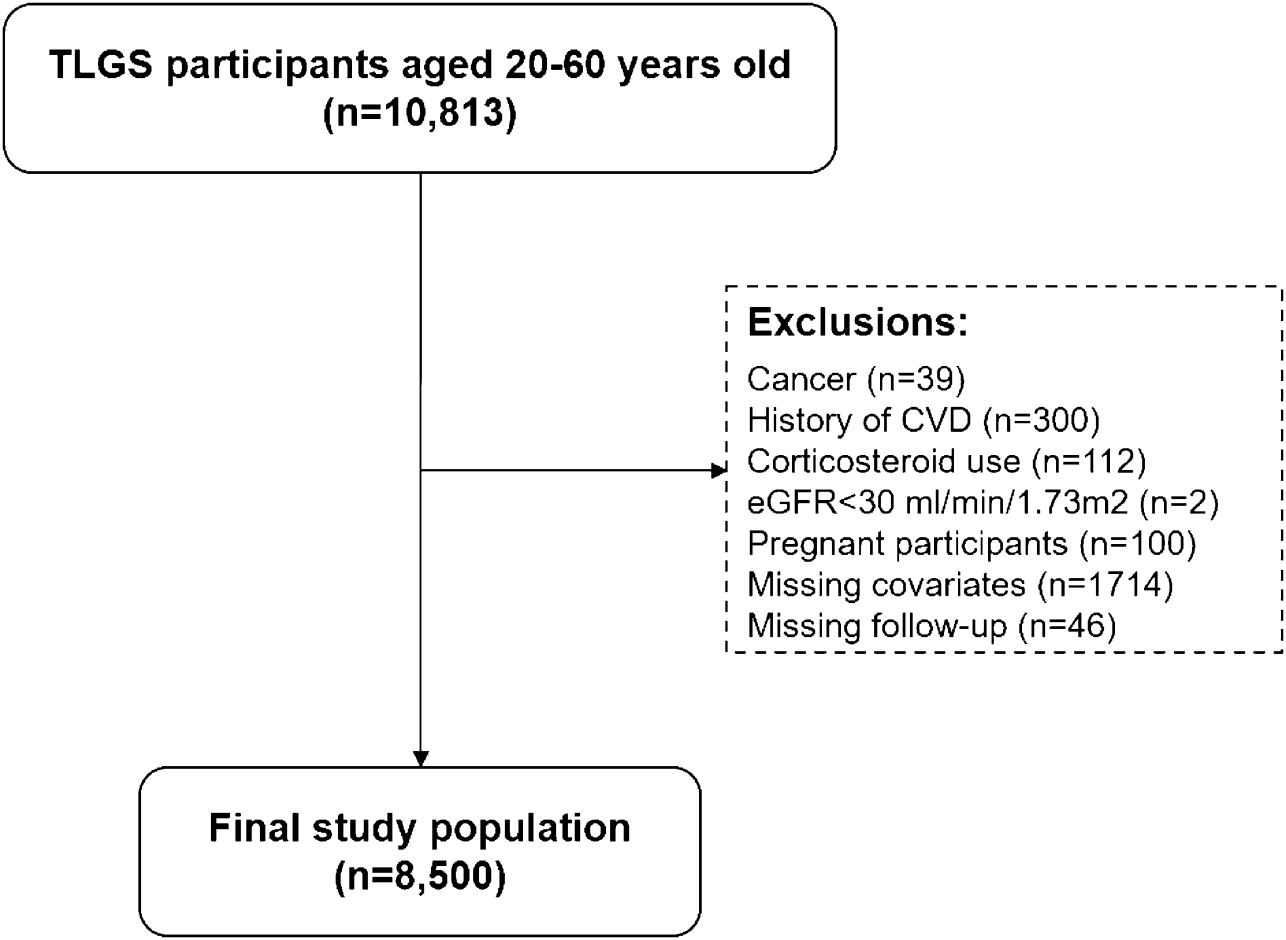
Flowchart illustrating participant selection. *TLGS:* Tehran Lipid and Glucose Study, *CVD:* Cardiovascular disease, *eGFR:* Estimated glomerular filtration rate.

Our study population includes participants from the Tehran Lipid and Glucose Study (TLGS), a 20-year large population-based cohort study. In brief, TLGS was first designed in 1999 to assess risk factors of non-communicable diseases and their prevalence in Iran. Detailed design of the TLGS has been published elsewhere^11^. For the current study, we included TLGS participants aged 20–60 years old at baseline. At baseline, individuals with cancer (n = 39), CVD (n = 300), use of systematic corticosteroids (n = 112), estimated glomerular filtration rate (eGFR) < 30 ml/min/1.73 m^2^ (n = 2), missing data (missing covariates: n = 1714; missing follow-up: n = 46), and pregnant participants (n = 100) were excluded from the study. Hence, a total of 8500 participants were enrolled and followed for 18 years (Fig. 1).

### Measurements and definitions

Demographic data and medical and drug history were collected through a pretested questionnaire. Smoking status was defined as the occasional or daily consumption of tobacco products. Education level was divided into three categories based on years of education or the degree they hold as follows: (1) Illiterate/primary school (< 6 years). (2) High school (6–12 years). (3) Higher education (≥ 12 years). Diagnosis of CVD in at least one first-degree relative before 55 years in men and 65 years in women was considered to have a positive family history of CVD. The data on physical activity were collected using the Persian version of the Lipid Research Clinic (LRC) questionnaire. Physical activity was defined using the metabolic equivalent of task scale (METS), by which individuals with METS < 600 min-week ^−1^ were categorized in the low physically active group. Weight and height were measured while patients were with minimal clothes and without shoes to the nearest 0.1 kg and 0.1 cm^2^ using standard protocols. Using a taped meter, WC was measured at the level of the umbilicus and recorded to the nearest 0.1 cm. Using a standardized mercury sphygmomanometer (calibrated by the Iranian Institute of Standards and Industrial Researches), systolic and diastolic blood pressure were measured twice after a 15-min rest, from the right arm, in the sitting position and the means were recorded as the participant's blood pressure.

After 12–14 h of fasting, blood samples were drawn from all the participants and were analyzed at the TLGS research laboratory on the day of blood collection. FPG, TG, HDL-C, and total cholesterol were measured using enzymatic colorimetric methods. Creatinine was assayed using the kinetic colorimetric Jaffe. All samples were analyzed when internal quality control met the acceptance criteria. Hypertension was defined as systolic blood pressure (SBP) ≥ 140 mmHg, diastolic blood pressure (DBP) ≥ 90 mmHg or the use of anti-hypertensive drugs. Dyslipidemia was defined as TG ≥ 150 mg/dL, TC ≥ 200 mg/dL, HDL-C < 40 mg/dL in men and HDL-C < 50 mg/dL in women, or the use of lowering lipid medication. The eGFR was calculated using the Chronic Kidney Disease Epidemiology Collaboration (CKD-EPI) 2021 formula^12^.

According to the joint interim statement (JIS) definition, MetS is defined as having three or more of the five following components: (1) WC ≥ 90 cm in both sexes according to the population-specific cut-off presented by the national committee^13^, (2) serum HDL-C < 40 mg/dl for men and < 50 mg/dl for women. (3) TG ≥ 150 mg/dl or specific treatment, (4) FPG ≥ 100 mg/dl, and (5) SBP ≥ 130 mmHg, DBP ≥ 85 mmHg or use of anti-hypertensive medication. In the international diabetes federation (IDF) criteria, the definition is based on the presence of abdominal obesity and two of the four remaining MetS components^14^.

### The cMetS-S definition

We have previously developed and validated age- and sex-specific cMetS-S score equations using CFA in each age and sex group separately (Supplementary Table S1)^10^. Briefly, this approach considers contributed weight of the traditional MetS components and their variations across age and sex subgroups. The cMetS-S can be calculated using the data of age, sex, and the MetS components’ values. Higher cMetS-S indicates higher MetS severity. In the current study, the cMetS-S was standardized at mean = 0 and SD = 1 based on the study population for straightforward interpretation and for generalizability of the results.

### Study outcomes

Included participants were followed annually for any medical events over 18 years follow-up. A consulting committee consisting of specialists, an epidemiologist, and the physician that registered the data, reviewed and evaluated the collected data to assign each individual an outcome. CVD events, including coronary and non-coronary heart disease (CHD and non-CHD; ICD-10 rubric codes I20-I25, I46.1, and I60-I69), all-cause and CVD mortality, and sudden cardiac death (SCD; I46.1) were selected as desired outcomes of the study. According to ICD-10, CHD events (I20-I25) comprise, (1) cases of definite or probable myocardial infarction with positive electrocardiogram findings plus cardiac signs and/or biomarkers, (2) cases going through any cardiac procedures (angiography-proven CHD with ≥ 50% stenosis in at least one major coronary artery, history of angioplasty or bypass surgery), and (3) cases with unstable angina pectoris (participants with new cardiac symptoms or change of pattern in previous cardiac symptoms and positive ECG findings with normal biomarkers and admitted to cardiac care unit).

### Statistical analysis

Baseline characteristics of the participants are reported as mean ± SD for continuous and frequency (percentage) for categorical variables. The comparison between the groups was made using an independent sample t-test for continuous and Chi-square test for categorical variables. The incident rate for the outcomes was calculated by dividing the total number of incident cases by the overall follow-up duration, measured in person-years. The association of cMetS-S with CVD and mortality was analyzed using continuous and tertile approaches with Cox proportional hazard regression models. Model 1 was adjusted for age and sex. Model 2 was adjusted for age, sex, smoking status, physical activity, CVD family history, and obesity (BMI ≥ 30 kg/m^2^). Model 3 was adjusted for model 2 and anti-hypertensive, anti-diabetic, and lipid-lowering medications. Model 4 was additionally adjusted for MetS components (high WC, high SBP, high TG, low HDL-C, and high FPG). We used restricted cubic spline regression analysis to demonstrate the relationship between cMetS-S and CVD and mortality. We also compared the model performance of JIS and IDF-based MetS and cMetS-S for predicting CVD and mortality using the Akaike information criterion (AIC) and log-likelihood ratio. The statistical analyses were performed using STATA 14 (StataCorp, college station, TX, USA) and R-3.0.3 (R Foundation for Statistical Computing, Vienna, Austria). A two-sided *P* value < 0.05 was considered statistically significant.

### Ethical approval and consent to participate

This study conformed to the ethical guidelines of the Helsinki Declaration and approved by the Human Research Review Committee of the Endocrine Research Center of Shahid Beheshti University of Medical Sciences, Tehran, Iran (IR.SBMU.ENDOCRINE.REC.1401.085). All participants provided informed written consent.

## Results

Baseline characteristics of the study population are presented according to cMetS-S tertiles in Table 1. Overall, we enrolled 8500 subjects (41.9% male) with a mean age of 37.4 ± 10.8 years. Approximately 32.2% and 27.9% of the participants were labeled as having MetS according to the JIS and IDF definitions; the MetS prevalence was higher in upper cMetS-S tertiles. Mean age, BMI, WC, lipid indices (except for HDL), SBP, and DBP increased toward upper cMetS-S tertiles (*P* < 0.001). Also, the prevalence of individuals who were smokers, low physically active, and used anti-hypertensive, anti-diabetic, and lipid-lowering drugs were higher in the higher cMetS-S tertiles (*P* < 0.001), while there was no difference in the prevalence of CVD family history across the tertiles.

**Table 1:** Baseline characteristics of the study population according to continuous metabolic syndrome severity score (cMetS-S) tertiles.

Characteristics	Total	cMetS-S tertiles			*P* value
		Tertile 1 (− 2.82, − 0.48)	Tertile 2 (− 0.49, 0.42)	Tertile 3 (0.43, 4.57)	
Number of participants	8500	2834	2833	2833	
Age (years)	37.4 ± 10.8	31.9 ± 9.5	38.0 ± 10.3	42.3 ± 10.0	< .001
Male n(%)	3562 (41.9)	853 (30.1)	1249 (44.1)	1460 (51.5)	< .001
Body mass index (kg/m^2^)	26.7 ± 74.7	23.60 ± 3.8	26.9 ± 4.0	29.5 ± 4.4	< .001
Waist circumference (cm)	87.2 ± 12.1	77.6 ± 9.2	87.8 ± 9.3	96.0 ± 9.9	< .001
Education n(%)					< .001
Illiterate/primary school (< 6 yrs.)	5394 (63.4)	1744 (61.5)	1817 (64.1)	1833 (64.7)	
High school (6–12 yrs.)	1820 (21.4)	583 (20.6)	614 (21.7)	623 (21.9)	
Higher education (≥ 12 yrs.)	1286 (15.1)	507 (17.9)	402 (14.2)	377 (13.3)	
Smokers n(%)	1222 (14.4)	296 (10.4)	427 (15.1)	499 (17.6)	< .001
Low physical activity n(%)	5967 (70.2)	1950 (68.8)	1967 (69.4)	2050 (72.3)	< .001
Family history of CVD n(%)	1379 (16.2)	385 (13.6)	462 (16.3)	532 (18.8)	< .001
Hypertension n(%)	1300 (15.3)	99 (3.5)	371 (13.1)	830 (29.3)	< .001
Dyslipidemia n(%)	3764 (44.3)	25 (0.88)	1088 (38.4)	2651 (93.6)	< .001
SBP (mmHg)	115.4 ± 15.8	107.5 ± 11.6	114.9 ± 13.8	123.8 ± 17.2	< .001
DBP (mmHg)	76.9 ± 10.4	71.8 ± 8.7	76.7 ± 9.4	81.8 ± 10.4	< .001
FPG (mg/dL)	94.7 ± 27.9	85.7 ± 8.7	90.3 ± 12.92	108.1 ± 42.5	< .001
Triglyceride (mg/dL)	164.8 ± 114.3	81.3 ± 23.9	142.1 ± 36.0	270.9 ± 136.4	< .001
Total cholesterol (mg/dL)					
HDL-C (mg/dL)	41.8 ± 10.8	48.6 ± 10.8	41.2 ± 9.1	35.7 ± 8.1	< .001
Anti-hypertensive medication n(%)	242 (2.8)	15 (0.53)	71 (2.5)	156 (5.5)	< .001
Anti-diabetic medication n(%)	148 (1.7)	4 (0.14)	15.0(0.53)	129 (4.5)	< .001
Lipid-lowering medication n(%)	136 (1.6)	7 (0.25)	25 (0.88)	104 (3.7)	< .001
MetS (JIS)	2735 (32.2)	22 (0.78)	465 (16.4)	2248 (79.3)	< .001
MetS (IDF)	2379 (27.9)	19 (0.67)	376 (13.3)	1984 (70.0)	< .001

The categorical and continuous variables were reported as count (percentage) and mean ± SD, respectively.

*cMetS-S* Continuous metabolic syndrome severity score, *SD* Standard deviation, *n* Number, *SBP* Systolic blood pressure, *DBP* Diastolic blood pressure, *FPG* Fasting plasma glucose, *HDL-C* High-density lipoprotein cholesterol, *MetS* Metabolic syndrome, *JIS* Joint Interim Statement, *IDF* International Diabetes Federation.

**Figure 2:**
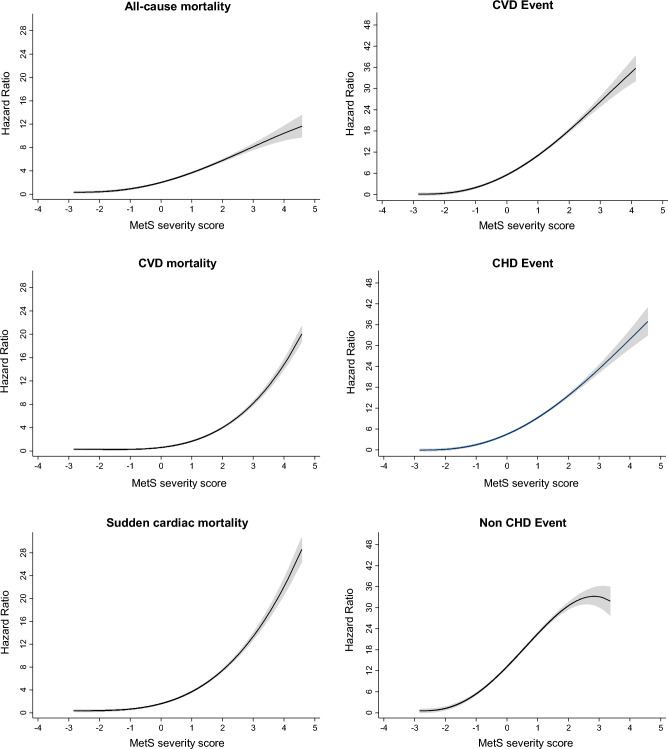
Restricted cubic spline regression analysis demonstrating the association of continuous metabolic syndrome severity score (cMetS-S) with all-cause, CVD, and sudden cardiac death (left panels), and CVD, CHD and non-CHD event (right panels). Heavy central lines represent the estimated fully adjusted hazard ratios in logarithmic scale, with shaded ribbons denoting 95% confidence intervals for each outcome. *CVD* Cardiovascular disease, *CHD* Coronary heart disease.

During 18 years of follow-up, 754 CVD incidents (524 CHD and 230 non-CHD events) and 292 all-cause mortality incidents (94 CVD and 198 non-CVD mortality) occurred. Incidence rates of CVD, mortality, and their subtypes increased incrementally across the cMetS-S tertiles (Tables 2, 3). The restricted cubic spline regression plots were generated according to the fully adjusted HRs obtained from the Cox proportional hazard model of participants for CVD and mortality. A non-linear positive association was found between cMetS-S and CVD and mortality outcomes (Fig. 2).

**Table 2:** Association of cMetS-S with cardiovascular events using continuous and tertile approach.

	No. of events	IR (95% CI)^a^	Model 1	Model 2	Model 3	Model 4
			HR (95% CI)	HR (95% CI)	HR (95% CI)	HR (95% CI)
CVD event						
CMetS-S^b^	749	6.0 (5.6–6.4)	1.67 (1.55–1.80)	1.68 (1.56–1.81)	1.60 (1.48–1.73)	1.67 (1.47–1.89)
Tertile						
Tertile 1	61	1.4 (1.1–1.8)	1.0 (Reference)	1.0 (Reference)	1.0 (Reference)	1.0 (Reference)
Tertile 2	207	5.0 (4.3–5.7)	2.09 (1.57–2.78)	2.09 (1.57–2.78)	2.03 (1.52–2.71)	2.03 (1.49–2.76)
Tertile 3	481	12.1(11.1–13.2)	3.76 (2.86–4.94)	3.75 (2.86–4.91)	3.42 (2.58–4.51)	3.39 (2.31–4.96)
CHD event						
CMetS-S^b^	521	4.2 (3.8–4.5)	1.69 (1.54–1.84)	1.69 (1.55–1.85)	1.63 (1.48–1.79)	1.60 (1.37–1.86)
Tertile						
Tertile 1	40	0.9 (0.7–1.2)	1.0 (Reference)	1.0 (Reference)	1.0 (Reference)	1.0 (Reference)
Tertile 2	140	3.4 (2.8–4.0)	2.10 (1.47–2.99)	2.10 (1.48–3.00)	2.08 (1.46–2.97)	1.96 (1.34–2.86)
Tertile 3	341	8.6 (7.7–9.5)	3.91 (2.80–5.46)	3.92 (2.81–5.47)	3.72 (2.64–5.22)	3.22 (2.03–5.13)
Non-CHD event						
CMetS-S^b^	228	1.9 (1.7–2.2)	1.72 (1.50–1.96)	1.71 (1.49–1.96)	1.59 (1.37–1.84)	1.88 (1.50–2.35)
Tertile						
Tertile 1	21	0.5 (0.3–0.7)	1.0 (Reference)	1.0 (Reference)	1.0 (Reference)	1.0 (Reference)
Tertile 2	67	1.7 (1.3–2.1)	2.10 (1.28–3.45)	2.08 (1.27–3.42)	1.91 (1.16–3.14)	2.15 (1.26–3.67)
Tertile 3	140	3.9 (3.3–4.6)	3.68 (2.30–5.90)	3.61 (2.26–5.78)	2.94 (1.81–4.79)	3.84 (1.96–7.51)

Model 1: adjusted for age and sex. Model 2: adjusted for age, sex, education, smoking, physical activity, CVD family history, and obesity (BMI ≥ 30 kg/m^2^). Model 3: adjusted for age, sex, education, smoking, physical activity, CVD family history, obesity (BMI ≥ 30 kg/m^2^), anti-hypertensive medication, anti-diabetic medication, and lipid-lowering medication. Model 4: adjusted for age, sex, education, smoking, physical activity, CVD family history, and individual metabolic syndrome components, including high waist circumference, high blood pressure, high triglycerides, low levels of HDL-C cholesterol, and high fasting plasma glucose.

*cMetS-S:* Continuous metabolic syndrome severity score, *HR:* Hazard ratio, *CI:* Confidence interval, *CVD:* Cardiovascular disease, *CHD:* Coronary heart disease.

^a^*IR* Incidence rate per 1000 person-years.

^b^Continuous analysis (per 1-SD increase) of cMetS-S.

**Table 3:** Association of cMetS-S with all-cause, cardiovascular, and sudden cardiac death using continuous and tertile approach.

	No. of events	IR (95% CI)^a^	Model 1	Model 2	Model 3	Model 4
			HR (95% CI)	HR (95% CI)	HR (95% CI)	HR (95% CI)
All-cause mortality						
CMetS-S^b^	291	2.2 (1.9–2.5)	1.35 (1.19–1.52)	1.35 (1.19–1.52)	1.24 (1.09–1.41)	1.37 (1.11–1.69)
Tertile						
Tertile 1	36	0.8 (0.6–1.1)	1.0 (Reference)	1.0 (Reference)	1.0 (Reference)	1.0 (Reference)
Tertile 2	95	2.2 (1.8–2.7)	1.53 (1.04–2.26)	1.53 (1.04–2.25)	1.50 (1.02–2.22)	1.54 (1.01–2.36)
Tertile 3	160	3.7 (3.2–4.4)	1.85 (1.27–2.68)	1.84 (1.27–2.66)	1.62 (1.10–2.39)	1.65 (0.93–2.90)
Cardiovascular disease mortality						
CMetS-S^b^	94	0.7 (0.6–0.9)	1.68 (1.37–2.06)	1.69 (1.38–2.07)	1.51 (1.21–1.88)	1.72 (1.20–2.45)
Tertile						
Tertile 1	3	0.1 (0.0–0.2)	1.0 (Reference)	1.0 (Reference)	1.0 (Reference)	1.0 (Reference)
Tertile 2	28	0.7 (0.4–0.9)	5.15 (1.51–16.9)	5.12 (1.54–16.9)	4.92 (1.43–16.2)	5.54 (1.60–18.9)
Tertile 3	63	1.5 (1.1–1.9)	8.15 (2.53–26.2)	8.25 (2.62–26.4)	6.83 (2.11–22.2)	9.50 (2.41–37.9)
Sudden cardiac death						
CMetS-S^b^	63	0.5 (0.4–0.6)	1.57 (1.22–2.03)	1.58 (1.23–2.03)	1.43 (1.09–1.88)	1.60 (1.03–2.49)
Tertile						
Tertile 1	2	0.0 (0.0–0.2)	1.0 (Reference)	1.0 (Reference)	1.0 (Reference)	1.0 (Reference)
Tertile 2	21	0.5 (0.3–0.7)	5.99 (1.49–25.6)	5.88 (1.41–25.2)	5.55 (1.34–23.8)	6.06 (1.31–27.2)
Tertile 3	40	0.9 (0.7–1.3)	8.22 (1.91–34.4)	8.23 (1.97–34.3)	6.63 (1.52–28.2)	8.68 (1.62–47.2)

Model 1: adjusted for age and sex. Model 2: adjusted for age, sex, education, smoking, physical activity, CVD family history, and obesity (BMI ≥ 30 kg/m^2^). Model 3: adjusted for age, sex, education, smoking, physical activity, CVD family history, obesity (BMI ≥ 30 kg/m^2^), anti-hypertensive medication, anti-diabetic medication, and lipid-lowering medication. Model 4: adjusted for age, sex, education, smoking, physical activity, CVD family history, and individual metabolic syndrome components, including high waist circumference, high blood pressure, high triglycerides, low levels of HDL-C cholesterol, and high fasting plasma glucose.

*cMetS-S:* Continuous metabolic syndrome severity score, *HR:* Hazard ratio, *CI:* Confidence interval.

^a^*IR* Incidence rate per 1000 person-years.

^b^Continuous analysis (per 1-SD increase) of cMetS-S.

As shown in Table 2, cMetS-S was associated with future CVD events with an HR of 1.67 (95% CI 1.47–1.89) per 1-SD increase in cMetS-S. The results were congruent when calculating the risk of CVD event subtypes; the risk of CHD and non-CHD events increased with HRs of 1.60 (95% CI 1.37–1.86) and 1.88 (95% CI 1.50, 2.35) per 1-SD increase of cMetS-S, respectively. The risk of incident CVD (HR 3.39; 95% CI 2.31–4.96), CHD (HR 3.22; 95% CI 2.03–5.13), and non-CHD (HR 3.84; 95% CI 1.96–7.51) events increased in the highest tertile compared to the lowest tertile.

The cMetS-S had a significant association with all-cause mortality with an HR of 1.24 (95% CI 1.09–1.41) per 1-SD increase in cMetS-S after adjusting for confounding variables (Table 3); the result was consistent after further adjusting for individual MetS components, indicating that cMetS-S increased the risk of all-cause mortality independent of MetS components (HR 1.37; 95% CI 1.11–1.69). The risk of CVD mortality and SCD also increased upon a 1-SD increment of cMetS-S (CVD mortality, HR 1.72; 95% CI 1.20–2.45; SCD, HR 1.60; 95% CI 1.03–2.49) in the fully adjusted model. The risk of all-cause mortality (HR 1.65; 95% CI 0.93–2.90), CVD (HR 9.50; 2.41–37.9), and SCD (HR 8.68, 95% CI 1.62–47.2) increased upon higher cMetS-S tertile in the fully adjusted model (Table 3).

We also examined whether cMetS-S has added value over MetS definitions and its components in predicting CVD and mortality independent of its components using model fit analysis (Tables 4, 5). The initial model was built based on MetS components (high TG, high WC, high FPG, high SBP, and low HDL-C) and the risk factors as covariates. We then separately added cMetS-S, MetS defined by JIS, and MetS defined by IDF to the initial model. Unlike JIS and IDF-based MetS definitions, cMetS-S performed better than the initial model by decreasing the AIC value by 24.1 and 59 on df = 1 for mortality and CVD prediction, respectively (*P* < 0.001). The association of JIS- and IDF-based definition with CVD and mortality are presented in Supplementary Tables S2 and S3.

**Table 4:** Model fit analysis of metabolic syndrome indicators for prediction of cardiovascular events.

	Variables in the model	− 2 log ${\hat{\text{L}}}$	AIC
Model (1)	Age + sex + education + physical activity + family history of CVD + anti-hypertensive medication + lipid-lowering medication + anti-diabetic medication + high WC + high BP + high TG + high HDL + high FPG	12,148.6	12,172.7
Model (2)	Model 1 + cMetS-S	12,089.6	12,115.6
Model (3)	Model 1 + MetS (JIS)	12,148.2	12,174.2
Model (4)	Model 1 + MetS (IDF)	12,148.7	12,174.7

Interpretation: Model (2) versus model (1): 12,148.6–12,089.6 = 59 on 1 df. This is significant (*P* < 0.001); therefore, there is evidence that adding cMetS-S to the CVD initial prediction model improves the model fit. Model (3) versus model (1): 12,148.6–12,148.2 = 0.4 on 1 df. This is non-significant (*P* = 0.52); therefore, there is evidence that MetS, defined by JIS, does not improve the initial model that includes MetS components. Model (4) versus model (1): 12,148.7–12,148.6 = 0.1 on 1 df. This is non-significant (*P* = 0.74); therefore, there is evidence that MetS, defined by IDF, does not improve the initial model that includes MetS components.

*CVD:* Cardiovascular disease, *WC:* Waist circumference, *BP:* Blood pressure, *TG:* Triglyceride, *HDL:* High density lipoprotein cholesterol, *FPG:* Fasting plasma glucose, *cMetS-S:* Continuous metabolic syndrome severity score, *JIS:* Joint Interim Statement, *IDF:* International Diabetes Federation, − *2 log* $\hat{L}$ Log-likelihood ratio, *AIC:* Akaike information criterion.

**Table 5:** Model fit analysis of metabolic syndrome indicators for prediction of mortality.

	Variables in the model	− 2 log ${\hat{\text{L}}}$	AIC
Model (1)	Age + sex + education + physical activity + family history of CVD + anti-hypertensive medication + lipid-lowering medication + anti-diabetic medication + high WC + high BP + high TG + high HDL + high FPG	4765.9	4789.9
Model (2)	Model 1 + cMetS-S	4741.8	4767.8
Model (3)	Model 1 + MetS (JIS)	4765.8	4791.8
Model (4)	Model 1 + MetS (IDF)	4765.2	4791.2

Interpretation: Model (2) versus model (1): 4741.8–4765.9 = 24.1 on 1 df. This is significant (*P* < 0.001); therefore, there is evidence that adding cMetS-S to the mortality initial prediction model improves the model fit. Model (3) versus model (1): 4765.8–4765.9 = 0.1 on 1 df. This is non-significant (*P* = 0.72); therefore, there is evidence that MetS, defined by JIS, does not improve the initial model that includes MetS components. Model (4) versus model (1): 4765.8–4765.9 = 0.1 on 1 df. This is significant (*P* = 0.74); therefore, there is evidence that MetS, defined by IDF, does not improve the initial model that includes MetS components.

*CVD* Cardiovascular disease, *WC* Waist circumference, *BP* Blood pressure, *TG* Triglyceride, *HDL* High density lipoprotein cholesterol, *FPG* Fasting plasma glucose, *cMetS-S* Continuous metabolic syndrome severity score, *JIS* Joint Interim Statement, *IDF* International Diabetes Federation, − *2 log* $\hat{L}$ Log-likelihood ratio, *AIC* Akaike information criterion.

**Figure 3:**
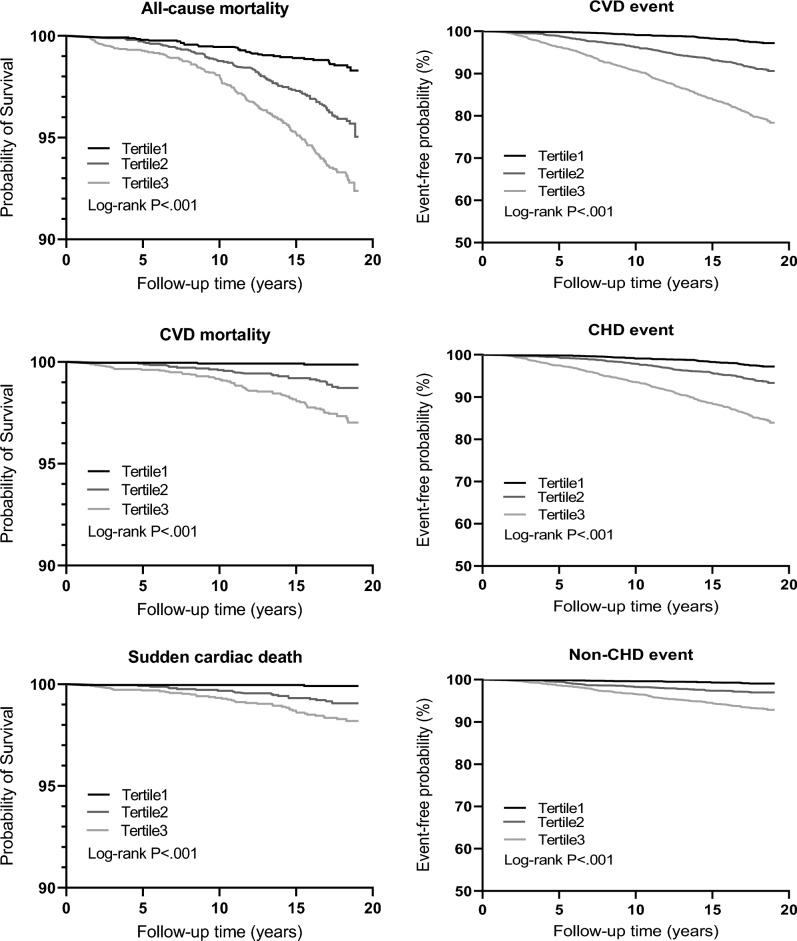
Kaplan–Meier survival curves showing event-free probability according to cMetS-S tertile for all-cause, CVD, sudden cardiac death (left panels), and CVD, CHD, and non-CHD event (right panels). *P* values from the log-rank test are displayed within the plots. *cMetS-S* Continuous metabolic syndrome severity score, *CVD* Cardiovascular disease, *CHD* Coronary heart disease.

The Kaplan–Meier curves demonstrated poorer event-free probability for individuals in upper tertiles of cMetS-S for CVD event and mortality outcomes (log *P* rank < 0.0001) (Fig. 3).

## Discussion

In the current study, we assessed the potential applicability of cMetS-S, an age- and sex-specific measure of MetS severity, to predict incident CVD and mortality during 18 years in a population-based cohort study. We found that cMetS-S was independently associated with CVD (CHD and non-CHD) and all-cause and CVD mortality among young and middle-aged populations using continuous and quantile approaches. The aforementioned association persisted even after controlling for MetS components. Additionally, our findings indicated that cMetS-S demonstrates a superior predictive ability for future CVD events and mortality compared to the conventional definitions of MetS based on JIS and IDF criteria.

The clinical utility of MetS as a predictive tool to assess the future risk of diabetes and CVD is limited due to its binary nature. No universal measure has been presented to define the severity of cardiometabolic abnormality based on MetS. Some researchers employ the number of MetS components as a metric for measuring MetS severity, thereby ignoring the extent of abnormality of the individual MetS components. A recent international multicenter study reported that the prevalence and the risk of future CVD were higher in individuals with MetS compared to those with a single MetS component; however, this risk was not elevated when compared to individuals with two MetS components^15^. This indicates the continuum nature of MetS and its attributed risk for CVD. The cMetS-S is a newly developed definition for MetS severity based on the values of traditional MetS components and their contribution to MetS on a continuous scale. In this newly developed definition for severity, the limitations in the traditional MetS criteria are overcome.

Recently, several useful phenotypes of preMetS and their corresponding indices have been defined. Studies demonstrated that hypertriglyceridemic-waist (HTGW) phenotype and triglyceride-glucose (TyG) index (representative of high FPG and TG phenotype) increased the risk of CVD events even after controlling for established risk factors^16–18^. The joint effect of high TG and FPG also provided an additional predictive ability for CVD incidence^19^. Although these phenotypes and indices can be helpful indicators of cardiometabolic health, their multiplicity creates confusion in health assessment at individual and public levels. The cMetS-S is a tangible quantitative measure that considers all these phenotypes and the degree of abnormality in MetS components. The cMetS-S provides a comprehensive health risk assessment for the whole population regardless of whether or not individuals meet the traditional definition of MetS.

In this study, we demonstrated that cMetS-S could well predict CVD incidence over long-term follow-up. Individuals with higher cMetS-S had a three times higher risk for CVD, CHD, and non-CHD events after adjusting for age, sex, education, physical activity, CVD family history, smoking, obesity, medications, and MetS components. This aligns with the findings of previous studies despite differences in confounding adjustments, methods, and study populations. In the study on the American population^8^, in contrast to the current and the Korean study^9^, the developed MetS severity was not formulated based on age categories; in addition, the adjustments were limited to age, sex, and individual MetS components. We also demonstrated a non-linear positive association between cMetS-S and CVD using continuous and tertile approaches. Previous studies only investigated this association using quantile classification and analysis^8,9^. We found that each SD increase in MetS severity leads to more than 60% increased risk of future CVD. Using a standardized continuous approach will allow for comparison and generalization of the results among different studies and populations and also prevent the loss of data due to quantile classification.

To the best of our knowledge, this is the first study to show the association between MetS severity derived from CFA and mortality. We found that cMetS-S is associated with an increased risk of all-cause, cardiovascular, and sudden cardiac death even after adjusting for MetS components. MetS is a complex and heterogeneous entity with variations in the pathophysiology, prevalence, and associated risk of individual MetS components for CVD and mortality across age and sex groups^20–22^. Using cMetS-S, we could overcome this limitation by defining MetS severity based on the contribution of MetS components to MetS in each sex and age subgroup. However, further research is needed to assess how defining MetS severity in different age and sex categories could improve the prediction of future adverse health outcomes.

MetS is a well-known risk factor for CVD, CHD, stroke, and mortality^23–25^; however, whether the traditional-based MetS definitions as a whole convey the same risk independent of its components is controversial^26–28^. Our findings suggest that unlike JIS and IDF-based definitions, cMetS-S has significant additional predictive value for CVD and mortality beyond MetS components. Our study is in line with the studies in the literature on American and Korean individuals showing cMetS-S to have an increased risk for CVD independent of the MetS components^8,9,29^. These findings indicate that MetS defined in continuous scale by CFA may better incorporate the complex interactions between metabolic processes involved in MetS by considering the weighted contribution of the MetS components.

The present study has several notable strengths. We validated the newly developed cMetS-S in a large, representative sample of Iranian adults with nearly two decades of follow-up. Additionally, this is the first study to evaluate cMetS-S utility for predicting all-cause, CVD, and sudden cardiac death. Furthermore, unlike the previous studies in the literature, we demonstrated the association of standardized MetS severity with CVD and mortality on a continuous scale (for each SD increase), allowing for comparison and generalization of the results from different studies. Moreover, age, sex, education, smoking, physical activity, CVD family history, obesity, medications, and individual MetS components were adjusted to reflect the independent association of MetS severity with CVD and mortality. Regarding our limitations, we restricted our participants to individuals aged between 20 and 60 years to investigate this association in the non-elderly population.

In conclusion, the newly developed age- and sex-specific MetS severity score is a reliable predictive tool for CVD and mortality among the Iranian population independent of MetS components. Unlike JIS and IDF-based MetS criteria, the cMetS-S provides additional value beyond MetS components in predicting CVD and mortality. The standardized cMetS-S has the potential to be presented as a new universal scoring system for MetS with respect to the variations in age, sex, and ethnicity.

### Supplementary Information

Supplementary Information.

## Data Availability

Datasets generated during and/or analyzed during the current study are not publicly available but are available from the corresponding author on reasonable request.

## Abbreviations

MetS: Metabolic syndrome
WC: Waist circumference
FPG: Fasting plasma glucose
TG: Triglyceride
HDL-C: High density lipoprotein cholesterol
CVD: Cardiovascular disease
CFA: Confirmatory factor analysis
cMetS-S: Continuous Mets severity score
TLGS: Tehran Lipid and Glucose Study
eGFR: Estimated glomerular filtration rate
METS: Metabolic equivalent of task scale
SBP: Systolic blood pressure
DBP: Diastolic blood pressure
CKD-EPI: Chronic Kidney Disease Epidemiology Collaboration
JIS: Joint interim statement
IDF: International Diabetes Federation
CHD: Coronary heart disease
AIC: Akaike information criterion
HTGW: Hypertriglyceridemic-waist
TyG: Triglyceride-glucose
