# Supplementary material for: Independent association between age- and sex-specific metabolic syndrome severity score and cardiovascular disease and mortality
Source: Sci Rep. 2023 Sep 5;13:14621. doi: 10.1038/s41598-023-41546-y (PMC10480156; doi:10.1038/s41598-023-41546-y)
Supplement: Supplementary file 1 — Supplementary Information. [file 41598_2023_41546_MOESM1_ESM.docx]

**Supplementary Table S1.** Age- and sex-specific equations of continuous metabolic syndrome severity score (cMetS-S) using confirmatory factor analysis

| **Sex** | **Age** | **Equations** |
| --- | --- | --- |
| Male | 20 – 39 yr. | -1.79 + 0.0016 × SBP + 0.0045 × WC + 0.0017 × FPG + 0.24 × ln (TG) - 0.0042× HDL-C |
|  | 40 – 60 yr. | -1.67 + 0.0007 × SBP + 0.0034 × WC + 0.0014 × FPG + 0.25 × ln (TG) - 0.0042× HDL-C |
| Female | 20 – 39 yr. | -2.43 + 0.0039 × SBP + 0.0066 × WC + 0.004 × FPG + 0.28 × ln (TG) - 0.0052 × HDL-C |
|  | 40 – 60 yr. | -2.37 + 0.001 × SBP + 0.0021 × WC + 0.0015 × FPG + 0.41 × ln (TG) - 0.004 × HDL-C |

Abbreviations: SBP, systolic blood pressure; WC, waist circumference; FPG, fasting plasma glucose; TG, triglyceride; HDL-C, high-density lipoprotein cholesterol

**Supplementary Table S2.** Association of JIS- and IDF-based defineition of metabolic syndrome with cardiovascular events

|  | **No. of Events** | **IR (95 % CI)^*^** | **Model 1** | **Model 2** | **Model 3** | **Model 4** |
| --- | --- | --- | --- | --- | --- | --- |
|  |  |  | **HR (95% CI)** | **HR (95% CI)** | **HR (95% CI)** | **HR (95% CI)** |
| **CVD Event** |  |  |  |  |  |  |
| MetS (JIS) |  |  |  |  |  |  |
| No (n = 5765) | 283 | 3.3 (2.9 - 3.7) | 1.0 (Reference) | 1.0 (Reference) | 1.0 (Reference) | 1.0 (Reference) |
| Yes (n=2735) | 466 | 12.1(11.1 - 13.3) | 2.09(1.79-2.43) | 2.09(1.79-2.43) | 1.87(1.59-2.19) | 1.47(1.18-1.84) |
| MetS (IDF) |  |  |  |  |  |  |
| No (n=6121) | 342 | 3.7 (3.3 - 4.1) | 1.0 (RE) | 1.0 (RE) | 1.0 (RE) | 1.0 (RE) |
| Yes (2379) | 407 | 12.2(11.1 - 13.4) | 1.91(1.65-2.21) | 1.90(1.64-2.20) | 1.73(1.47-2.03) | 1.39(1.16-1.66) |
| **CHD Event** |  |  |  |  |  |  |
| MetS (JIS) |  |  |  |  |  |  |
| No (n = 5765) | 189 | 2.2 (1.9 - 2.5) | 1.0 (Reference) | 1.0 (Reference) | 1.0 (Reference) | 1.0 (Reference) |
| Yes (n=2735) | 332 | 8.6 (7.8 - 9.6) | 2.23(1.85-2.68) | 2.23(1.86-2.68) | 2.06(1.70-2.51) | 1.59(1.22-2.07) |
| MetS (IDF) |  |  |  |  |  |  |
| No (n=6121) | 233 | 2.5 (2.2 - 2.9) | 1.0 (Reference) | 1.0 (Reference) | 1.0 (Reference) | 1.0 (Reference) |
| Yes (2379) | 288 | 8.6 (7.7 - 9.7) | 1.97(1.65-2.35) | 1.96(1.65-2.34) | 1.87(1.55-2.27) | 1.41(1.13-1.74) |
| **Non-CHD Event** |  |  |  |  |  |  |
| MetS (JIS) |  |  |  |  |  |  |
| No (n = 5765) | 94 | 1.1 (0.9 - 1.4) | 1.0 (Reference) | 1.0 (Reference) | 1.0 (Reference) | 1.0 (Reference) |
| Yes (n=2735) | 134 | 3.8 (3.2 - 4.5) | 1.91(1.45-2.51) | 1.90(1.45-2.50) | 1.54(1.15-2.07) | 1.27(0.85-1.89) |
| MetS (IDF) |  |  |  |  |  |  |
| No (n=6121) | 109 | 1.2 (1.0 - 1.5) | 1.0 (Reference) | 1.0 (Reference) | 1.0 (Reference) | 1.0 (Reference) |
| Yes (2379) | 119 | 3.9 (3.3 - 4.7) | 1.88(1.44-2.45) | 1.87(1.43-2.44) | 1.51(1.12-2.02) | 1.41(1.01-1.95) |

Model 1: adjusted for age and sex.
Model 2: adjusted for age, sex, education, smoking, physical activity, CVD family history, and obesity (BMI≥30kg/m^2^).
Model 3: adjusted for age, sex, education, smoking, physical activity, CVD family history, and obesity (BMI≥30kg/m^2^), anti-hypertensive medication, anti-diabetic medication, and lipid-lowering medication
Model 4: adjusted for age, sex, education, smoking, physical activity, CVD family history, and individual metabolic syndrome components, including high waist circumference, high blood pressure, high triglycerides, low levels of HDL-C cholesterol, and high fasting plasma glucose.
Abbreviations: JIS, joint interim statement; IDF, international diabetes federation; HR, hazard ratio; CI, confidence interval;*IR: Incidence rate per 1,000 person-years.

**Supplementary Table S3.** Association of JIS- and IDF-based definition of metabolic syndrome with mortality

|  | **No. of Events** | **IR (95 % CI)^*^** | **Model 1** | **Model 2** | **Model 3** | **Model 4** |
| --- | --- | --- | --- | --- | --- | --- |
|  |  |  | **HR (95% CI)** | **HR (95% CI)** | **HR (95% CI)** | **HR (95% CI)** |
| **All-cause mortality** |  |  |  |  |  |  |
| MetS (JIS) |  |  |  |  |  |  |
| No (n = 5765) | 133 | 1.5 (1.3 - 1.8) | 1.0 (Reference) | 1.0 (Reference) | 1.0 (Reference) | 1.0 (Reference) |
| Yes (n=2735) | 158 | 3.8 (3.3 - 4.5) | 1.36 (1.07-1.73) | 1.36 (1.07-1.72) | 1.17 (0.90-1.52) | 0.92 (0.64-1.31) |
| MetS (IDF) |  |  |  |  |  |  |
| No (n=6121) | 150 | 1.6 (1.4 - 1.9) | 1.0 (Reference) | 1.0 (Reference) | 1.0 (Reference) | 1.0 (Reference) |
| Yes (2379) | 141 | 3.9 (3.3 - 4.6) | 1.40 (1.10-1.77) | 1.39 (1.10-1.76) | 1.27 (0.98-1.64) | 1.13 (0.84-1.51) |
| **CVD mortality** |  |  |  |  |  |  |
| MetS (JIS) |  |  |  |  |  |  |
| No (n = 5765) | 33 | 0.4 (0.3 - 0.5) | 1.0 (Reference) | 1.0 (Reference) | 1.0 (Reference) | 1.0 (Reference) |
| Yes (n=2735) | 61 | 1.5 (1.1 - 1.9) | 2.11(1.37-3.27) | 2.16(1.40-3.34) | 1.76(1.10-2.81) | 1.65 (0.87-3.15) |
| MetS (IDF) |  |  |  |  |  |  |
| No (n=6121) | 39 | 0.4 (0.3 - 0.6) | 1.0 (Reference) | 1.0 (Reference) | 1.0 (Reference) | 1.0 (Reference) |
| Yes (2379) | 55 | 1.5 (1.2 - 2.0) | 2.06(1.36-3.14) | 2.10(1.38-3.20) | 1.78(1.12-2.81) | 1.69 (0.89-3.20) |
| **Sudden cardiac death** | |  |  |  |  |  |
| MetS (JIS) |  |  |  |  |  |  |
| No (n = 5765) | 23 | 0.3 (0.2 - 0.4) | 1.0 (Reference) | 1.0 (Reference) | 1.0 (Reference) | 1.0 (Reference) |
| Yes (n=2735) | 40 | 1.0 (0.7 - 1.3) | 2.11(1.24-3.59) | 2.15(1.27-3.65) | 1.75(0.99-3.08) | 1.90 (0.77-4.68) |
| MetS (IDF) |  |  |  |  |  |  |
| No (n=6121) | 26 | 0.3 (0.2 - 0.4) | 1.0 (Reference) | 1.0 (Reference) | 1.0 (Reference) | 1.0 (Reference) |
| Yes (2379) | 37 | 1.0 (0.7 - 1.4) | 2.19(1.31-3.67) | 2.24(1.34-3.74) | 1.84(1.05-3.22) | 1.82(0.98-3.55) |

Model 1: adjusted for age and sex.
Model 2: adjusted for age, sex, education, smoking, physical activity, CVD family history, and obesity (BMI≥30kg/m^2^).
Model 3: adjusted for age, sex, education, smoking, physical activity, CVD family history, and obesity (BMI≥30kg/m^2^), anti-hypertensive medication, anti-diabetic medication, and lipid-lowering medication
Model 4: adjusted for age, sex, education, smoking, physical activity, CVD family history, and individual metabolic syndrome components, including high waist circumference, high blood pressure, high triglycerides, low levels of HDL-C cholesterol, and high fasting plasma glucose.
Abbreviations: JIS, joint interim statement; IDF, international diabetes federation; HR, hazard ratio; CI, confidence interval;*IR: Incidence rate per 1,000 person-years.
